# Functional Heterogeneity of Breast Fibroblasts Is Defined by a Prostaglandin Secretory Phenotype that Promotes Expansion of Cancer-Stem Like Cells

**DOI:** 10.1371/journal.pone.0024605

**Published:** 2011-09-21

**Authors:** Jenny A. Rudnick, Lisa M. Arendt, Ina Klebba, John W. Hinds, Vandana Iyer, Piyush B. Gupta, Stephen P. Naber, Charlotte Kuperwasser

**Affiliations:** 1 Graduate Program in Cell, Molecular and Developmental Biology Program, Sackler School of Graduate Biomedical Sciences, Tufts University School of Medicine, Boston, Massachusetts, United States of America; 2 Molecular Oncology Research Institute, Tufts Medical Center, Boston, Massachusetts, United States of America; 3 Whitehead Institute for Biomedical Research, Broad Institute, Department of Biology, Massachusetts Institute of Technology and Harvard, Cambridge, Massachusetts, United States of America; 4 Department of Pathology, Tufts Medical Center, Boston, Massachusetts, United States of America; Massachusetts General Hospital, United States of America

## Abstract

Fibroblasts are important in orchestrating various functions necessary for maintaining normal tissue homeostasis as well as promoting malignant tumor growth. Significant evidence indicates that fibroblasts are functionally heterogeneous with respect to their ability to promote tumor growth, but markers that can be used to distinguish growth promoting from growth suppressing fibroblasts remain ill-defined. Here we show that human breast fibroblasts are functionally heterogeneous with respect to tumor-promoting activity regardless of whether they were isolated from normal or cancerous breast tissues. Rather than significant differences in fibroblast marker expression, we show that fibroblasts secreting abundant levels of prostaglandin (PGE2), when isolated from either reduction mammoplasty or carcinoma tissues, were both capable of enhancing tumor growth *in vivo* and could increase the number of cancer stem-like cells. PGE2 further enhanced the tumor promoting properties of fibroblasts by increasing secretion of IL-6, which was necessary, but not sufficient, for expansion of breast cancer stem-like cells. These findings identify a population of fibroblasts which both produce and respond to PGE2, and that are functionally distinct from other fibroblasts. Identifying markers of these cells could allow for the targeted ablation of tumor-promoting and inflammatory fibroblasts in human breast cancers.

## Introduction

Fibroblasts were first described in the late 19^th^ century by the pathologist Rudolph Virchow based on their residence within connective tissues and their elongated, spindle-like shape [1]. As the most prominent cell type within connective tissues, these mesenchymal cells function to deposit and remodel extracellular matrix (ECM), specify epithelial fate and maturation of tissues, facilitate granulation of tissues post wounding and promote re-epithelialization [2], [3], [4], [5], [6]. Fibroblasts are required for mammary gland development, as signals from the underlying primary mesenchyme are required to induce mammary placode elongation and invasion to form the primitive mammary ductal tree [7].

In addition to regulating and maintaining tissue homeostasis, fibroblasts are well established mediators of tissue fibrosis following injury and promoters of epithelial tumor growth. During fibrosis, the acquisition of epigenetic alterations [8] results in fibroblasts with altered gene expression, conferring an increase in growth factor production, ECM deposition, and proliferation [9]. During carcinoma progression, the associated desmoplastic stroma includes an abundance of alpha smooth muscle actin (αSMA+) expressing fibroblasts, collectively referred to as cancer associated fibroblasts (CAFs). These cells are also found in connective tissues during wound healing and are frequently termed “myofibroblasts”. These αSMA+ fibroblasts isolated from the stroma of solid tumors can significantly promote the growth of breast [10], [11], [12], prostate [13], pancreas [14], and skin cancer cells in mice [15]. Some of the tumor promoting mechanisms of these cells have been established, such as increasing tumor angiogenesis and mediating macrophage recruitment, both of which serve as a prominent sources of growth factors and cytokines for the growth of tumor cells [16]. Recently, αSMA+ myofibroblasts have also been shown to possess a pro-inflammatory phenotype [15], suggesting that these cells may contribute to the tumor associated inflammation that accompanies the progression of tumors.

Despite the established functions of fibroblasts in tissue homeostasis and disease, the molecular mechanisms contributing to the phenotypic and functional heterogeneity among fibroblasts remains largely unknown [17], [18], [19], [20], [21]. It has been reported that the gene expression of fibroblasts derived from disease- free breast tissue harbors a greater heterogeneity than those derived from breast carcinomas, even within the same patient [22]. This is surprising, given that breast tumors are heterogeneous and there exists significant heterogeneity among the expression of αSMA within the tumor associated stroma [23]. While αSMA is a common marker routinely used to identify CAFs, these cells may vary in the levels of αSMA expression [24], and it remains unknown if robust expression of αSMA marks the most tumor promoting fibroblasts within a given fibroblast population. It also should be noted that several cell types in the tumor microenvironment, in addition to fibroblasts, will express αSMA [25], [26], [27], and it is not a unique marker of CAFs or myofibroblasts. Moreover, it is well established that fibroblasts isolated from homeostatic tissues will acquire αSMA expression and stress fibers, resembling the phenotype of myofibroblasts when explanted in culture, exposed to TGFβ, or exposed to tumor cell conditioned media [28], [29], [30]. Because of this, maintaining the phenotypic and functional discrepancy of fibroblasts from homeostatic versus diseased tissues in culture has been extremely difficult.

Intriguingly, αSMA+ fibroblasts isolated from tumor associated stroma are not the only source of fibroblasts that harbor the capacity to support epithelial tumor growth. Fibroblasts isolated from arthritic synovium can promote growth of co-mixed human breast cancer cells in a xenograft mouse model of breast cancer [31]. However, there are numerous examples to demonstrate the tumor suppressive function of fibroblasts as well. For example, ras-transformed mouse keratinocytes are unable to form tumors in syngeneic animals when they are co-mixed with dermal fibroblasts isolated from disease-free mouse skin [32]; initiated primary human prostate epithelial cells do not form tumors under the renal capsule when co-mixed with fibroblasts from disease free human prostate tissues, unlike their CAF counterparts [13]. These functional discrepancies among fibroblast populations remain to be reconciled.

In this manuscript, we sought to understand the properties of breast fibroblasts that contribute to a tumor promoting phenotype. Using fibroblast populations isolated from both disease-free and breast tumor tissues, we demonstrate for the first time that the ability of fibroblasts to promote tumor growth is irrespective of tissue source and correlates with the ability of these cells to secrete PGE2 and respond to PGE2 signaling. These data may lend insight into the dichotomy among the tumor promoting or tumor suppressive functions of various fibroblast populations, and warrants the investigation of novel markers for these cells among heterogeneous fibroblast populations. Discovery of such markers may elucidate which patients in the clinic would largely benefit from an adjuvant therapy targeting both the eradication of tumor cells and the tumor promoting fibroblasts within the tumor stroma.

## Materials and Methods

### Isolation of primary fibroblasts from human breast tumors and reduction mammoplasty tissues

All human breast tissue procurement for these experiments was obtained in compliance with the laws and institutional guidelines, as approved by the institutional IRB committee from Tufts University School of Medicine. Primary breast tumor tissues were obtained from discarded material at Tufts Medical Center and non-cancerous breast tissue was obtained from patients undergoing elective reduction mammoplasty at Tufts Medical Center. Breast tissues were minced and enzymatically digested overnight with a mixture of collagenase and hyaluronidase as previously described [33], [34]. Large clusters of undigested tissue were allowed to settle and the supernatant enriched for stromal cells was collected, washed and plated in serum containing medium to enrich for mammary fibroblasts. Cells were grown in DMEM supplemented with 10% calf serum (CS) and antibiotic/antimycotic (AB/AM, Invitrogen, Carlsbad, CA) for multiple passages until senescent as described previously [34]. Senescent fibroblasts were not included in subsequent experiments.

### Cell cultures, conditioned media collection, and cell treatments

Fibroblasts were grown as described above. MCF7 breast cancer cells were grown in DMEM+10% CS+1% AB/AM. All cultures were maintained at 37 °C and 5% CO2. To generate conditioned media, fibroblasts were seeded at 1.5×10^6^ cells/plate in phenol red free DMEM (PRF-DMEM, Invitrogen), supplemented with either 0.5% or 2% charcoal/dextran stripped FBS (Invitrogen) and 1% AB/AM, treated with ethanol or 0.5 µM PGE2 (Sigma-Aldrich, St. Louis, MO), or left untreated, for 72 hrs. CM was then harvested, filtered through a 0.22 µm filter (Millipore, Danvers, MA), aliquoted and stored at −80°C. For MCF7 treatments, cells were grown in PRF-DMEM supplemented with 5% charcoal/dextran stripped FBS, 1% AB/AM, and treated with ethanol, 0.5 µM PGE2, or 1 nM 17-β-Estradiol (Sigma Aldrich). For MCF7 treatments involving fibroblast CM, CM was collected as previously described, supplemented with 5% charcoal/dextran stripped FBS, and administered to MCF7 cells for 6 days. α-IL6 and recombinant human IL-6 (R&D Systems, Minneapolis, MN) were used at 1.5 µg/mL and 10 ng/mL, respectively.

### Preparation of cells for mouse mammary fat pad inoculation

All animal procedures were performed in accordance with an approved protocol by Tufts University Institutional Animal Care and Use Committee. A colony of NOD/SCID mice was maintained under sterile housing conditions and received food and water *ad libum*. Nulliparous female mice between 8 to 14 weeks age were utilized in all experiments. For co-mixing experiments, 1.5×10^6^ human breast fibroblasts were mixed with 500,000 MCF7 breast cancer cells, resuspended in a 3∶1 (vol/vol) ratio of media to Matrigel (BD Biosciences, San Jose, CA) mixture, and inoculated into the 4^th^ inguinal mammary gland. Tumor formation was assessed by palpitation weekly. For MCF7 limiting dilution experiments, 500,000 MCF7 cells were cultured in conditioned media (CM) from PGE2-treated fibroblasts for 6 days, after which 10,000 cells were resuspended in a 3∶1 (vol/vol) ratio of media to Matrigel mixture, and inoculated into the 4^th^ inguinal mammary gland. Tumor formation was assessed by palpitation weekly.

### Immunofluorescence

Fibroblasts were seeded at 5,000 cells/well of 8 well-chamber slides (Becton Dickinson, Franklin Lakes, NJ) in DMEM+10% CS+1% AB/AM (Invitrogen) for 96 hours before fixed in methanol. Cells were then permeabilized with 0.1% Triton X-100 (Sigma) in PBS, washed and blocked in 1% BSA in PBS at ambient temperature. Cells were incubated with the following antibodies overnight at 4 °C: mouse α-alpha SMA (1∶200, Vector Labs, Burlingame, CA), mouse α-Vimentin (1∶200, Vector Labs), mouse α-prolyl-4-hydroxylase (1∶300, Millipore), and mouse α-caveolin-1 (1∶150, Novus Biologicals, Littleton, CO). Fluorescence signal was detected using goat α-mouse secondary antibodies (1∶500, conjugated with Alexa488 and Alexa588, Invitrogen). Nuclei were stained with 4′, 6-diamidino-2-phenylindole (DAPI) and images were captured with the Spot imaging software system (Diagnostic Instruments, Inc., Sterling Heights, MI). Quantification was performed using Image-J software.

### Quantitative Real Time PCR

RNA was isolated and purified using an RNeasy kit (Qiagen, Valencia, CA) for cultured cells. RNA was reverse transcribed to cDNA using an iScript cDNA synthesis kit (Biorad). Quantitative real time PCR analysis was performed using SyBR Green and an iCycler thermocycler (Biorad). Primer sequences used for quantitative real-time PCR are listed in Table S1.

### Flow cytometry

Nonconfluent cultures of MCF7 cells were trypsinized into single cell suspension, counted, and resuspended in FACS buffer (PBS+3% CS). 100,000 cells were stained with the following antibodies for 15 minutes at ambient temperature: α-human CD24-PE (BD Biosciences), α-human CD44-APC (BD Biosciences), α-human ESA-FITC (Stem Cell Technologies, Vancouver, BC, Canada), and isotype controls for each antibody (Mouse IgG_2a_-PE, Mouse IgG_2b_-APC, Mouse IgG_1_-FITC, BD Biosciences). Unbound antibody was washed away with FACS buffer, and cells were analyzed no longer than 1 hr post staining on a BD FACS Calibur.

### Tumorsphere assays

Fibroblasts were treated with either ethanol or 0.5 µM PGE2, and conditioned media was prepared as described above. Upon use, conditioned media was supplemented with 5% charcoal/dextran-stripped FBS. MCF7s were dissociated to a single cell suspension, plated at 20,000 cells/ml, and grown on ultra-low adherence 6-well plates (Corning Life Sciences, Lowell, MA) in the presence of supplemented conditioned media for 6 days. For recombinant human IL-6 studies or exogenous PGE2 studies, MCF7s were plated as described above in the presence of PRF-DMEM supplemented with 5% charcoal/dextran-stripped FBS and the following treatments: 0.1% BSA in PBS or 10 ng/mL recombinant human IL-6 (R&D Systems), or ethanol or 0.5 µM PGE2 (Sigma Aldrich). Tumorspheres were quantified using a Multisizer-3 coulter counter (Beckman-Coulter, Brea, CA).

### PGE2 enzyme immunoassay

Fibroblast conditioned media was prepared as described above and subjected to a Prostaglandin E2 monoclonal EIA kit according to the manufacturer's instructions (Cayman Chemical, Ann Arbor, MI). Concentration (pg/ml) was determined by generating a standard curve with known concentrations of PGE2. Absorbance was read at 405 nm using a 96 well plate μQuant spectrophotometer (Biotek, Winooski, VT) and the KC Junior software program (Biotek).

### Cytokine array

Fibroblasts were treated with either ethanol or 0.5 µM PGE2, and CM was prepared as described above. Human cytokine arrays (2000 series, RayBiotech, Norcross, GA) were exposed to CM isolated from ethanol or 0.5 µM PGE2-treated fibroblasts and processed according to the manufacturer's instructions. Exposed films were quantified for chemiluminescence intensity using a Xenogen phosphoimager.

### IL-6 ELISA

Fibroblast conditioned media was prepared as described above and subjected to a human specific IL-6 ELISA according to the manufacturer's instructions (eBiosciences, San Diego, CA). Concentration (pg/ml) was determined by generating a standard curve with known concentrations of recombinant human IL-6. Absorbance was read at 450 nm using a 96 well plate μQuant spectrophotometer (Biotek, Winooski, VT) and the KC Junior software program (Biotek). Concentrations were normalized to total numbers of fibroblasts after 72 hour exposure to either ethanol or 0.5 µM PGE2 and reported as arbitrary units.

### Western blotting

MCF7 cells were seeded at 250,000 cells/well and exposed to CM or treatments as described above. After 6 days, cells were pelleted and lysed in RIPA buffer to prepare cell lysates. Fibroblast conditioned media was prepared as previously described and concentrated using.

Amicon Ultra Centrifugal Filters (Millipore). Protein concentration from both lysates and conditioned media was determined using a Lowry Assay according to manufacturer's instructions (Biorad, Hercules, CA). 30 or 50 µg of protein was resolved on a 4–12% polyacrylamide gel, transferred to a nitrocellulose membrane (Biorad), and immunoblotted using the following antibodies: mouse α-GAPDH (1∶5000, Millipore), mouse α-phosphorylated-STAT3 (Y705, 1∶1000, Cell Signaling Technologies, Danvers, MA), rabbit α-total-STAT3 (1∶2000, Cell Signaling Technologies), mouse α-Cox-2 (Cayman Chemical), mouse α-Cox-1 (Cayman Chemical), or rabbit α-IL6 (1∶10,000, Abcam, Cambridge, MA). Signal was detected using HRP-conjugated goat α-mouse (1∶14,000) or goat α-rabbit (1∶12,000) secondary antibodies (Cell Signaling) and West Dura Extended Chemiluminescence Substrate (Fisher).

### Immunohistochemistry

Tumor tissues from MCF7 xenografts were fixed in 10% neutral buffered formalin and paraffin embedded with standard procedures. Tumor sections were deparaffinized, re-hydrated through graded ethanols and subjected to heat-induced antigen retrieval. Staining was performed by the Department of Pathology Core Facility at Tufts Medical Center.

## Results

### Isolation and characterization of fibroblasts from human breast tissues

We obtained fibroblasts from a variety of resected breast tissue samples (Table 1): those derived from breast tumor specimens of varying hormone receptor and HER2 status, and those derived from disease-free reduction mammoplasty tissues. Given the inability to prospectively sort for different populations of fibroblasts due to the lack of established and unique cell surface markers for these cells, we used the ability of fibroblasts to preferentially adhere to plastic and grow *ex-vivo* under defined conditions. Quantitative RT-PCR and immunofluorescence were used to characterize and verify that the cells isolated from human breast tissues were indeed enriched in fibroblasts.

**Table 1:** Summary of tissue derived fibroblasts used for this study.

Patient ID	Tissue Source	αSMA Expression	Basal IL-6 Secretion	PGE2 Secretion
**A**	Mastectomy; ER+/HER2−/BRCA1 carrier	Medium	Low	Medium
**C**	Mastectomy; ER−/HER2−	Low	Low	High
**D**	Invasive lobular carcinoma; ND	ND	Low	ND
**E**	Invasive ductal carcinoma; ER−/PR−/HER2+	Medium	Medium	Medium
**F**	Lumpectomy; ND	ND	ND	Medium
**G**	Mastectomy; ER+/HER2−	ND	ND	ND
**H**	Phyllodes tumor	ND	ND	ND
**I**	Mammoplasty; disease free	High	Low	Low
**J**	Mammoplasty; disease free	ND	Medium	Medium
**K**	Mammoplasty; disease free	Low	Low	High
**L**	Mammoplasty; disease free	ND	Medium	ND
**M**	Mammoplasty; disease free	Low	Medium	Medium
**N**	Mammoplasty; disease free	Medium	ND	ND

αSMA expression is defined as: low, <45% positive; medium, 45–55% positive; high, >55% positive. ND, not determined.

**Figure 1:**
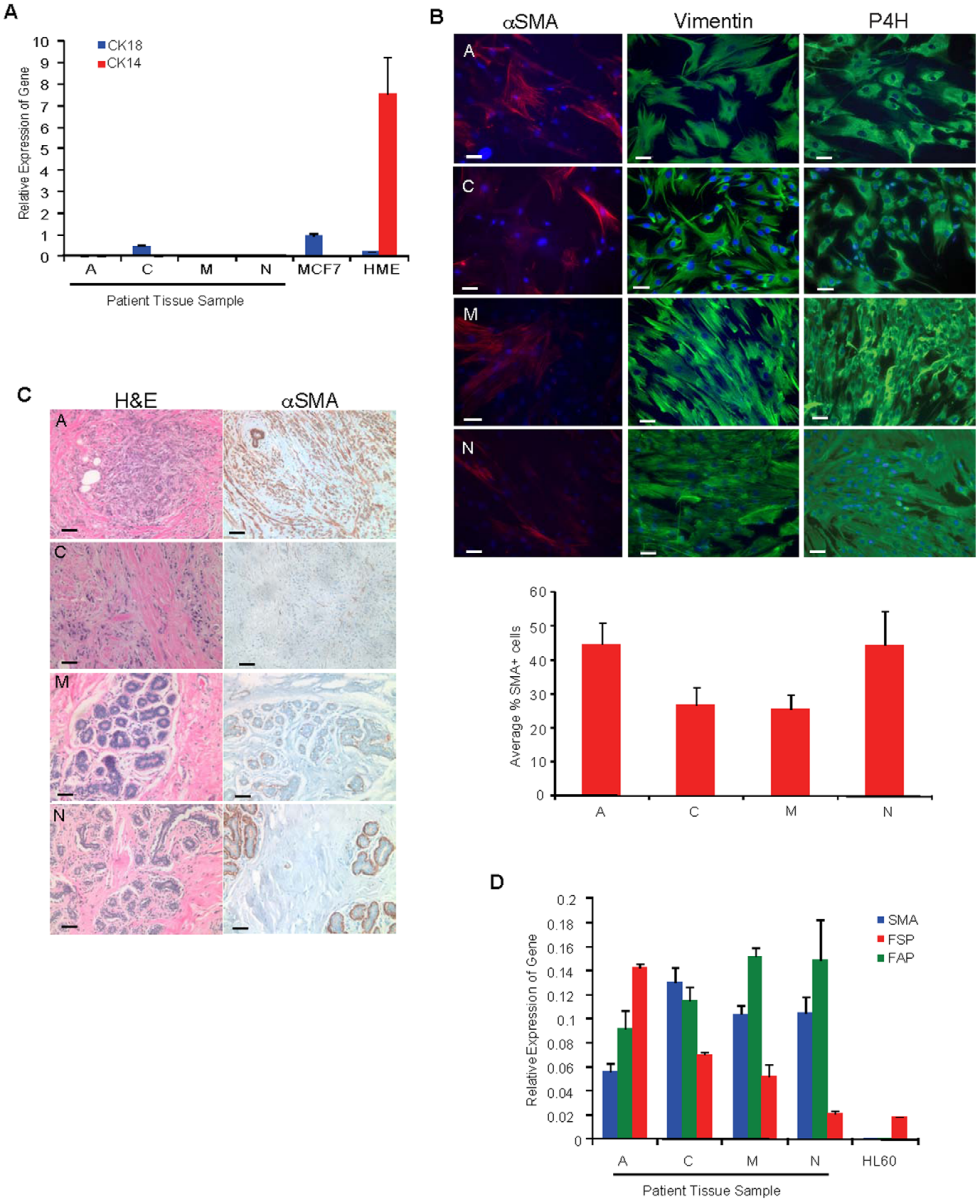
Characterization of patient derived fibroblasts from human breast tumor tissues and reduction mammoplasty tissues. (**A**) Quantitative RT-PCR for the relative levels of breast epithelial markers CK18 and CK14 transcripts in tissue derived fibroblasts from patient samples A, C, M and N. MCF7 and immortalized human mammary epithelial cells (HME) serve as positive controls for CK18 and CK14, respectively. (**B**), Top, immunofluorescence for the expression of mesenchymal markers vimentin and prolyl-4-hydroxylase (P4H) in tissue-derived fibroblasts from patient samples A, C, M and N. Nuclei are stained with DAPI. Scale bar, 50 µm. Bottom, quantification of the percentage of αSMA+ cells per total number of cells in a given field. (**C**), H&E stains and αSMA immunohistochemistry of human breast tumor tissue sections (A, C) and human reduction mammoplasty tissue sections (M, N) from which fibroblasts were derived. Scale bar, 50 µm. (**D**) Quantitative RT-PCR for the relative levels of αSMA, FAP, and FSP transcripts in tissue-derived fibroblasts from patient samples A, C, M and N after propagating *in vitro*. HL60 cells are shown as a negative control. Statistics were performed with a single factor ANOVA, p = 0.143).

We first assayed for the expression of fibroblast and epithelial markers to ascertain the purity of the cultured stromal cells. Cells were largely negative for cytokeratin 18 (CK18) and cytokeratin 14 (CK14) expression (Fig. 1A, Fig. S1B), indicating these cells were devoid of breast epithelial cells. All patient derived fibroblasts robustly expressed vimentin and prolyl-4-hydroxylase (P4H) (Fig. 1B and Fig. S1A). In addition to these fibroblast markers, we also assayed for the expression of αSMA, a marker of myofibroblasts and cancer associated fibroblasts (CAFs). Consistent with previous reports, αSMA expression *in vivo* was only present within the stroma associated with breast tumors but not within the stroma of the normal human breast tissues (Fig. 1C). However, cultured fibroblasts isolated from all the different patient samples acquired αSMA expression as previously reported [29], [35], [36]; expression was similar among patient derived fibroblasts despite differences in expression *in vivo*. Interestingly, none of the patient derived fibroblasts, regardless of the tissue source of origin, were more than 50% αSMA positive as measured by immunofluorescence (Fig. 1B); there was no significant difference in αSMA expression among these patient samples (Fig. 1B, p = 0.1433). We corroborated these immunofluorescence results by quantitative RT-PCR (Fig. 1D). Moreover, to avoid the confounding effects of serum-induced αSMA expression in cultured cells, we serum starved patient derived fibroblasts for 96 hours. Even under serum starvation conditions for this extended period of time, the levels in αSMA expression between these patient samples did not change (data not shown).

Recently, fibroblast surface protein (FSP) and fibroblast activation protein (FAP) have been reported to distinguish different types of fibroblasts in tumor tissues. Therefore, we also examined FSP and FAP expression in breast tissue derived fibroblasts. Both CAFs and disease-free breast fibroblasts harvested from several different patient samples expressed high transcript levels of these two proteins (Fig. 1D).

Given that αSMA, FSP or FAP expression *in vitro* could not distinguish fibroblasts isolated from human breast tumor specimens versus fibroblasts isolated from disease-free human breast tissues, we sought to identify other proteins whose expression may be enriched in one tissue source or the other. Recently, expression of Caveolin-1 has been reported to be downregulated in CAFs compared to disease-free fibroblasts, and loss of Caveolin-1 in mammary stromal fibroblasts promotes a tumor promoting, CAF-like phenotype [37], [38], [39]. However, we found high expression of Caveolin-1 in fibroblasts from both breast tumor specimens and disease-free tissues, indicating that, similar to αSMA, this protein could not distinguish between these cell types *in vitro* (Fig. S1A).

### An inflammatory phenotype correlates with fibroblast tumor promoting ability

**Figure 2:**
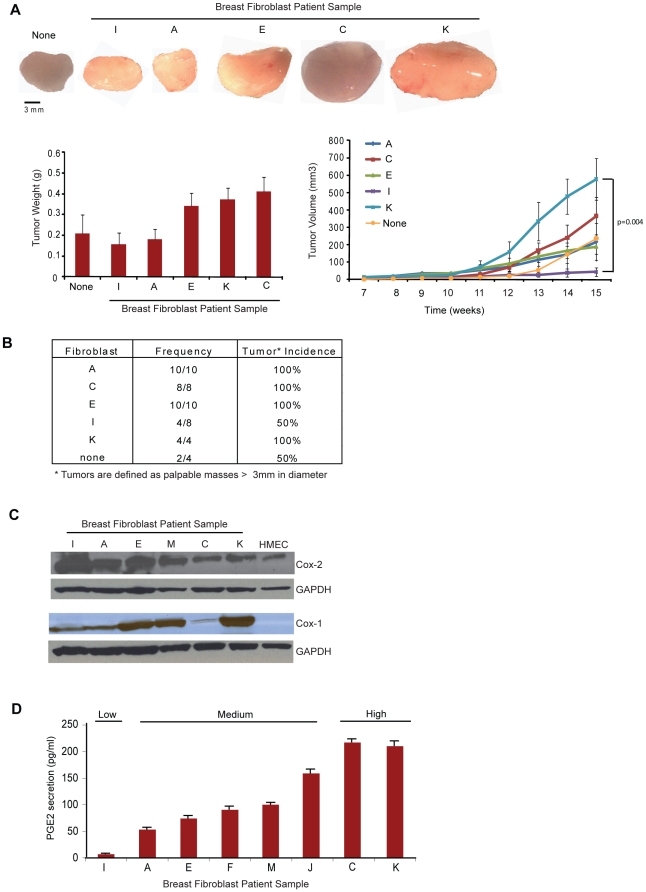
Fibroblast mediated tumor promotion *in vivo* correlates with PGE2 secretion *in vitro*. (**A**) Resected tumors from mice inoculated with a co-mix of tissue-derived fibroblasts from 5 different patients (A, C, E, I, K) and MCF7 breast cancer cells, or MCF7 cells injected alone (none). Scale bar, 3 mm. Bottom left, tumor weights from mice inoculated with a co-mix of tissue-derived fibroblasts from 5 different patients (A, C, E, I, K) and MCF7 breast cancer cells, or MCF7 cells injected alone (none). Statistics were performed using a single factor ANOVA, p = 0.045). Bottom right, average tumor volume (mm^3^) from mice inoculated with a co-mix of tissue-derived fibroblasts from 5 different patients (A, C, E, I, K) and MCF7 breast cancer cells, or MCF7 cells injected alone (none), assessed over 16 weeks. Statistics were performed using a Kruskal-Wallis nonparametric ANOVA (p = 0.02) followed by a two-tailed t test of means, comparing the volume of tumors derived from MCF7s co-mixed with patient sample K to those derived from MCF7s co-mixed with patient sample I (p = 0.004). Error bars, SEM. (**B**) Tumor incidence from mice inoculated with a co-mix of tissue-derived fibroblasts from 5 different patients (A, C, E, I, K) and MCF7 breast cancer cells, or MCF7 cells injected alone (none). (**C**) Cox-2 and Cox-1 western blots of lysates prepared from various tissue-derived fibroblasts. Human mammary epithelial cells (HMECs) are shown as a positive control for Cox-2. (**D**) PGE2- based immunoassay of CM from various tissue-derived fibroblasts. PGE2 concentrations are determined by a set of control standards of known concentration, according to the manufacturer's instructions. Statistics were performed using a single factor ANOVA (p = 2.84×10^−12^). Error bars, SEM.

Given the lack of specific marker expression between fibroblasts isolated from breast tumor tissues versus fibroblasts isolated from disease-free breast tissues, we sought to assay for functional differences in promoting mammary tumor growth *in vivo*. Accordingly, we co-mixed fibroblasts from 5 different tissue sources (patients A, C, E, I and K) with weakly tumorigenic, estrogen-dependent MCF7 cells, and inoculated ad-mixed cells into the inguinal mammary fat pad of female NOD/SCID mice. Fibroblasts derived from tissue samples C, K, and E co-mixed with MCF7 cells formed larger tumors than MCF7 cells injected alone, whereas fibroblasts derived from tissue sample I failed to support MCF7 tumor growth (p = 0.02, Fig. 2A), and only supported tumor formation 50% of the time (Fig. 2B). Thus, tumor promoting ability of fibroblasts did not depend on their tissue source of origin (i.e. breast tumor or reduction mammoplasty, Table 1), nor did it associate with their extent of αSMA, FSP, FAP, or Caveolin-1 expression (Fig. 1 and Fig. S1A).

Based on these findings, we sought to identify characteristics of these tissue derived fibroblasts that could resolve the differences in their ability to support MCF7 tumor growth. Recent reports have indicated that CAFs express an NFκB mediated pro-inflammatory secretome that is required for their ability to support tumor growth [15]. Thus, we hypothesized that fibroblasts differ in their inherent pro-inflammatory state, which might account for their tumor promoting capabilities *in vivo*. In fact, all tissue-derived fibroblasts *in vitro*, regardless of their tissue of origin, expressed cyclooxygenase-2 (Cox-2), an enzyme upregulated during tissue inflammation and tumorigenesis [40], as well as its isoform Cox-1 (Fig. 2C and Fig. S2). Both Cox-2 and Cox-1 catalyze the synthesis of the pro-inflammatory hormone prostaglandin E2 (PGE2), although Cox-1 is thought to be constitutively expressed in most tissues and likely responsible for producing the levels of prostaglandins required for normal tissue function [41].

Given that these tissue-derived fibroblasts express Cox-1 and Cox-2, we assayed for the levels of PGE2 in the conditioned media (CM) from these cells. Fibroblasts from 8 different tissue samples were serum starved for 72 hrs in phenol-red free DMEM, upon which the CM was collected, filtered, and assayed for the concentration of the pro-inflammatory hormone PGE2 by a specific immunoassay. CM from several different tissue samples exhibited significant differences in PGE2 secretion (p = 2.84×10^−12^): low (<50 pg/ml; patient sample I), medium (50–150 pg/ml; tissue samples A, E, F, M, J) or high (>200 pg/ml; tissue samples C, K) (Fig. 2D). Intriguingly, we found a correlation between the ability to secrete high levels of PGE2 *in vitro* and the ability to promote MCF7 tumor growth *in vivo*. In fact, there was a statistically significant difference in the ability to support MCF7 tumor growth between high PGE2 secreting fibroblasts (K) compared to low PGE2 secreting fibroblasts (I) (p = 0.004). These findings suggest that unlike αSMA, FAP, FSP, or Caveolin-1 expression, the ability to secrete PGE2 may contribute to the tumor promoting phenotype of fibroblasts.

### Secreted factors from tumor-promoting fibroblasts expand breast cancer stem-like cells

**Figure 3:**
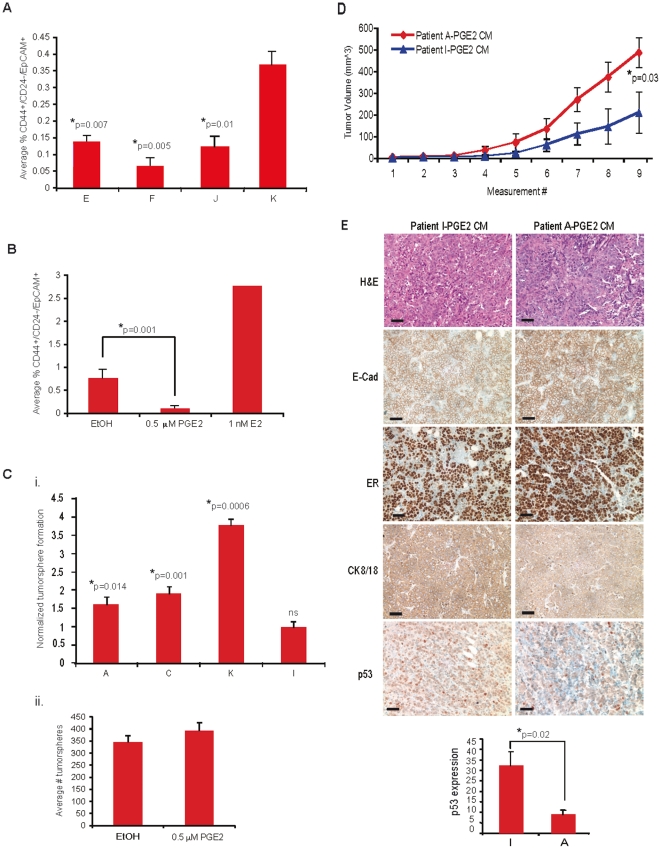
PGE2 enhances the tumor promoting properties of breast tissue-derived fibroblasts. (**A**) CM from various patient derived fibroblasts (E, F, J, K) were used to culture MCF7s for 6 days. The average percentage of CD44^+^/CD24^−^/EpCAM^+^ cells was then assayed by FACS. Statistics were performed using a single factor ANOVA (p = 0.007) followed by a Student's two-tailed t test of means: E vs. K, p = 0.007; F vs. K, p = 0.005; J vs. K, p = 0.01. Error bars, SEM. (**B**) MCF7 cells were cultured in either PRF-DMEM+10% CD-FBS supplemented with 1 nM E2 (positive control), 0.5 µM PGE2, or vehicle for 6 days. The average percentage of CD44^+^/CD24^−^/EpCAM^+^ cells was assayed by FACS. Data is plotted as average of 3 independent experiments. Statistics were performed using a Student's two tailed t test of means: EtOH vs. PGE2, p = 0.001. Error bars, SEM. (**C**) Quantification of MCF7 tumorspheres formed in the presence of CM from PGE2- or EtOH- fibroblasts from patient samples A, C, K, and I. Quantification was performed using a Beckman Coulter Multisizer and plotted as a fold induction over vehicle. Statistics were performed using a single factor ANOVA (p = 3.25×10^−5^) followed by a Student's two-tailed t test of means comparing EtOH vs PGE2 for each patient sample: A, p = 0.014; C, p = 0.001; K, p = 0.0006; I, not significant). Error bars, SEM. (**D**), Tumor growth curves of 10^4^ MCF7 cells primed *in vitro* with CM from PGE2-treated fibroblasts from patient sample A (A-PGE2 CM) or CM from PGE2-treated fibroblasts from patient sample I (I-PGE2 CM). Statistics were generated using a Student's two-tailed t-test of means; p = 0.03. Error bars, SEM (**E**) Top, H&E stains and immunohistochemical analysis (E-cadherin, ER, CK8/18, p53) of tumors (200×). Scale bar, 50 µm. Bottom, quantification of the percentage of p53 positive cells within a given field based on the tumor sections from (**D**) Statistics were performed using a Student's two-tailed t-test of means, p = 0.02).

It is known that breast tumors are comprised of cells with varying tumorigenic potential. Using primary human breast cancer tissues, it has been demonstrated that the CD44^+^/CD24^−^/EpCAM^+^ population of cells can support tumor initiation with as few as 100 cells and have self-renewing, stem-like properties [42]. These “aggressive” cell populations are retained within commonly used breast cancer cell lines, with MCF7 cells having a basal percentage of 0.03% [33], [43]. Because high PGE2-secreting fibroblasts support MCF7 tumor growth, we hypothesized that these fibroblasts may secrete factors that expand the most aggressive, tumorigenic cells within the tumor bulk. To determine if fibroblasts could expand this aggressive cell population, we treated MCF7 cells for 6 days with CM isolated from serum starved tissue-derived fibroblasts exhibiting varying degrees of PGE2 secretion *in vitro*. We quantified the percentage of aggressive, CD44^+^/CD24^−^/EpCAM^+^ stem-like cells by FACS following this 6-day treatment. CM from fibroblasts of varying tumor promoting potential and PGE2 secretion had significant differences in their ability to expand the percentage of aggressive MCF7 cells (Fig. 3A, p = 0.0007). Interestingly, those fibroblasts exhibiting high levels of PGE2 secretion resulted in a significant increase in the proportion of aggressive MCF7 cells compared to those fibroblasts that had medium or low secretion of PGE2 *in vitro* (E vs K, p = 0.007; F vs K, p = 0.005; J vs K, p = 0.01). Moreover, the ability to expand CD44^+^/CD24^−^/EpCAM^+^ cells was associated with the ability to support MCF7 tumor growth *in vivo* (Fig. 3A and Fig. 2A, respectively).

Recently, it has been reported that PGE2 is necessary for the expansion of bone marrow hematopoietic stem cells [44]. Since fibroblasts exhibiting high levels of PGE2 secretion also exhibited the most robust increase in cancer stem-like cells and promoted the largest MCF7 tumor formation, we reasoned that paracrine production of PGE2 by fibroblasts might be sufficient to expand CD44^+^/CD24^−^/EpCAM^+^ MCF7 stem-like cells. Therefore, we treated MCF7 cells with 0.5 µM PGE2, 1 nM 17-β-estradiol (E2, positive control), or EtOH (vehicle) for 6 days, and quantified the percentage of CD44^+^/CD24^−^/EpCAM^+^ cells by FACS. We found, however, that PGE2 alone was not sufficient to expand aggressive MCF7 cells (Fig. 3B).

Given the striking association between the ability of fibroblasts to secrete PGE2 *in vitro* and the ability to expand CD44^+^/CD24^−^/EpCAM^+^ cells and support tumor growth, we speculated that perhaps PGE2 signaling enhances the tumor promoting phenotypes of fibroblasts. To test this hypothesis, we chose to examine the CM from fibroblast patient samples who did not robustly enhance the growth of MCF7 breast cancer cells *in vivo* (patient samples A and I, Fig. 2A). First, we confirmed that these tissue-derived fibroblasts express prostanoid receptors EP1, EP2, EP3 and EP4, making them sensitive to PGE2 signaling (Fig. S2B). Next, we exposed these tissue-derived fibroblasts to exogenous 0.5 µM PGE2 or EtOH (vehicle) for 72 hours before harvesting CM. We then exposed MCF7 cells to this CM for 6 days before quantifying the population of CD44^+^/CD24^−^/EpCAM^+^ cells by FACS. Interestingly, exposure to PGE2 before CM collection significantly augmented the ability of tissue sample A to increase the percentage of CD44^+^/CD24^−^/EpCAM^+^ cells (Fig. S3), suggesting that PGE2 enhanced fibroblast tumor promoting ability. However, exposure to PGE2 did not significantly enhance the ability of tissue sample I to increase expansion of CD44^+^/CD24^−^/EpCAM^+^ cells (Fig. S3), despite prostanoid receptor expression (Fig. S2B).

We also performed *in vitro* and *in vivo* functional assays of MCF7 tumorgenicity using CM from PGE2- or vehicle (EtOH) treated fibroblasts. Specifically, MCF7 cells were grown under non-adherent culture conditions to promote tumorsphere formation in the presence of CM from PGE2- or vehicle (EtOH) treated fibroblasts of varying tumor promoting capability and endogenous PGE2 secretion status. As expected, CM from PGE2 treated tissue derived fibroblasts (tissue samples A, C and K), formed significantly more tumorspheres than those treated with CM from vehicle treated fibroblasts (A, p = 0.014; C, p = 0.001; K, p = 0.0006; Fig. 3C). However, CM from fibroblasts derived from tissue sample I did not significantly enhance MCF7 tumorsphere formation when primed with exogenous PGE2 (Fig. 3C), consistent with the inability to further enhance CD44^+^/CD24^−^/EpCAM^+^ cell expansion (Fig. S3B). Importantly, 0.5 µM PGE2 was not sufficient to enhance MCF7 tumorsphere formation compared to vehicle control (Fig. 3C).

We also sought to demonstrate that fibroblasts primed with PGE2 promote the aggressiveness of MCF7 cells *in vivo*. To this end, we exposed MCF7 cells first *in vitro* to CM from PGE2-treated tissue sample A, or CM from PGE2-treated tissue sample I, for 6 days to enrich for aggressive CD44^+^/CD24^−^/EpCAM^+^ cells. After 6 days, 10^4^ cells from each cohort were inoculated into the inguinal mammary gland of NOD/SCID mice. MCF7 cells exposed *in vitro* to CM from PGE2-treated tissue sample A formed significantly larger tumors than those exposed *in vitro* to CM from PGE2-treated tissue sample I (p = 0.03, Fig. 3D). Histological and immunohistochemical analysis of these tumors revealed that the tumors were all ERα- and E-cadherin-positive with a significant degree of central necrosis, (Fig. 3E). Interestingly, tumors derived from MCF7 cells exposed *in vitro* to CM from PGE2-treated tissue sample A exhibited reduced p53 and CK8/18 expression compared to those tumors formed from MCF7 cells exposed to CM from PGE2-treated tissue sample I (Fig. 3E, p = 0.02). Together, these data suggest that PGE2 signaling in fibroblasts enhances their tumor promoting abilities, resulting in expansion of aggressive tumor cell populations and increased tumor growth.

### PGE2 mediated IL-6 secretion by tumor-promoting fibroblasts

Since PGE2 alone was not sufficient to confer expansion of aggressive cancer stem-like cells (Fig. 3A), we hypothesized factors produced from PGE2-stimulated fibroblasts were essential for this expansion. To identify the secreted proteins mediating breast cancer stem-like expansion, we examined the CM from PGE2-treated or vehicle-treated tissue derived fibroblasts from patient A, and quantitatively assayed for 164 secreted growth factors and cytokines using an antibody-based protein array. We observed that the secretion of IL-6 was increased at least 5 fold upon PGE2-treatment compared to vehicle-treated control (Fig. S4A).

**Figure 4:**
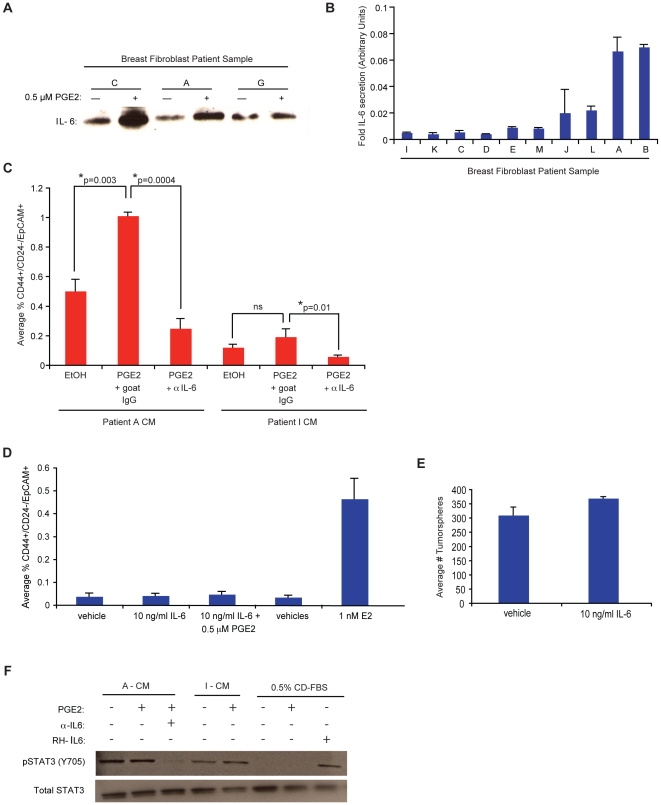
Tissue-derived fibroblasts secrete IL-6, which is necessary, but not sufficient, for the expansion of CD44^+^/CD24^−^/ESA^+^ cells. (**A**) Immunoblot for IL-6 expression in CM from tissue derived fibroblasts (from patient samples C, A, G) exposed to either EtOH (vehicle) or 0.5 µM PGE2. (**B**) CM from various tissue derived fibroblasts treated with either 0.5 µM PGE2 or EtOH (vehicle) were assayed for the levels of IL-6 secretion using a human IL-6 ELISA. Concentration is normalized to the cell number post treatment and plotted as the geometric mean of the fold induction (in arbitrary units). Error bars, SEM. (**C**) Quantification of the average percentage of CD44^+^/CD24^−^/EpCAM^+^ MCF7 cells after 6 day exposure to CM from PGE2 or EtOH treated fibroblasts from patient sample A (A-CM), or CM from PGE2 or EtOH treated fibroblasts from patient sample I (I-CM) in the presence of either goat IgG (control) or 1.5 µg/ml IL-6 neutralizing antibody. Statistics were performed using a Student's two-tailed t test of means: A-CM, EtOH vs. PGE2, p = 0.003; A-CM, PGE2 vs. α-IL6, p = 0.0004; I-CM, EtOH vs. PGE2, not significant. Error bars, SEM. (**D**) Quantification of the average percentage of CD44^+^/CD24^−^/EpCAM^+^ MCF7 cells after 6 day exposure to either 0.1% BSA (vehicle) or 10 ng/ml recombinant human IL-6, alone or in combination with 0.5 µM PGE2 or EtOH (vehicle). Error bars, SEM. (**E**) Quantification of MCF7 tumorspheres formed in the presence of DMEM+0.1% BSA (vehicle) or 10 ng/ml IL-6. Quantification was performed using a Multisizer-3 coulter counter. (**F**) Western blot of pSTAT3 and total STAT3 in lysates of MCF7 cells exposed for 30 min to CM from fibroblasts from patient A (A-CM) or fibroblasts from patient I (I-CM) treated with either EtOH, PGE2, or PGE2 plus 1.5 µg/ml α-IL6. MCF7 cells exposed for 30 min to DMEM+EtOH, PGE2 or 10 ng/ml IL-6 are shown as negative and positive controls for pSTAT3, respectively.

To more comprehensively assess whether PGE2 treated fibroblasts secrete IL-6, we performed both qualitative and quantitative assays. We first isolated CM from PGE2 or vehicle treated fibroblasts from 3 patient derived tissue samples (C, A, and G), concentrated the CM, and immunoblotted for IL-6 (Fig. 4A). Nearly all patient samples showed an increase in IL-6 secretion upon exposure to PGE2, supporting the cytokine array results. For a more quantitative assessment of IL-6 secretion, we performed a human specific IL-6 ELISA with CM prepared from various breast tissue derived fibroblasts treated with either 0.5 µM PGE2 or EtOH (vehicle) for 3 days. Although the fold induction of IL-6 secretion in response to PGE2 was heterogeneous among tissue derived fibroblasts, nearly all patient samples showed at least a 1.5 fold increase in IL-6 secretion upon treatment with PGE2 (Fig. 4B). However, the induced secretion of IL-6 by PGE2 (Fig. 4B), as well as the basal levels of IL-6 secretion in these tissue derived fibroblasts (Fig. S4B) both failed to associate with the tumor promoting abilities of these fibroblasts *in vivo* (Fig. 2A), or their ability to secrete PGE2 *in vitro* (Fig. 2C). Collectively, these data suggest that unlike high PGE2 secretion, high basal IL-6 secretion or PGE2 mediated induction of IL-6 secretion, are not necessarily hallmarks of tumor promoting fibroblasts.

### IL-6 is necessary but not sufficient for tumor-promoting fibroblasts to expand stem-like cells

It has been reported that IL-6 enhances aggressive, stem-like features in sphere-forming populations of human mammary epithelial cells [45], and mediates a positive feedback loop that maintains oncogenic transformation [46]. Thus, we suspected it might be an essential cytokine produced by fibroblasts that is required for expansion of CD44^+^/CD24^−^/EpCAM^+^ cells. To test this, we exposed MCF7 cells to CM from PGE2- or vehicle (EtOH) treated fibroblasts from tissue sample A and tissue sample I for 6 days in the presence of an IL-6 neutralizing antibody (αIL-6) or goat IgG control. Quantifying the percentage of CD44^+^/CD24^−^/EpCAM^+^ cells by FACS revealed that PGE2 significantly enhanced the ability of fibroblasts from tissue sample A to support expansion of CD44^+^/CD24^−^/EpCAM^+^ cells (p = 0.003, Fig. 4C) in an IL-6 dependent manner, as the expansion was attenuated in the presence of an IL-6 neutralizing antibody (αIL-6, p = 0.0004). PGE2 did not enhance the ability of fibroblasts from tissue sample I to support CD44^+^/CD24^−^/EpCAM^+^ cell expansion, despite some IL-6 present in the CM (Fig. 4C).

Given that IL-6 is necessary for fibroblast mediated expansion of CD44^+^/CD24^−^/EpCAM^+^ cells, we asked if IL-6 was sufficient to confer this expansion. We treated MCF7 cells with 10 ng/mL recombinant human IL-6 or 0.1% BSA (vehicle), in either the presence or absence of 0.5 µM PGE2 or EtOH (vehicle). Interestingly, IL-6 alone, or in combination with 0.5 µM PGE2, was not sufficient to expand CD44^+^/CD24^−^/EpCAM^+^ MCF7 cells, unlike 1 nM E2 (Fig. 4D). Also, IL-6 alone was not sufficient to confer increases in MCF7 tumorsphere formation (Fig. 4E). To confirm that MCF7 cells were capable of signaling through IL-6, we exposed MCF7 cells for 30 min to CM from PGE2 or EtOH treated fibroblasts of differing tumor-promoting capabilities, in the presence of an IL-6 neutralizing antibody or goat IgG control, and immunoblotted for the levels of phosphorylated STAT3 as an indicator of the IL-6 signaling pathway. pSTAT3 levels were higher in MCF7 cells exposed to CM from PGE2-treated fibroblasts from patient sample A as compared to CM from PGE2-treated fibroblasts from tissue sample I, and this was abrogated by addition of αIL-6. Moreover, 0.5 µM PGE2 was not sufficient to induce pSTAT3 expression, unlike the addition of 10 ng/mL recombinant human IL-6 (Fig. 4F). Collectively, these data indicate that IL-6 is enriched in CM from several patient tissue-derived fibroblasts, secreted by fibroblasts in response to PGE2, and is necessary, but not sufficient, for fibroblast-mediated expansion of CD44^+^/CD24^−^/EpCAM^+^ MCF7 cells.

## Discussion

Several studies have implicated fibroblasts as potent tumor promoting stromal cells through their ability to modulate the tumor microenvironment [16] and to promote the growth of cancer cells [10], [13], [14], [15], [47]. However, there also exists substantial evidence that fibroblasts from disease-free tissues have potent tumor suppressive properties [48]. The characteristics of these fibroblasts that contribute to either a tumor promoting or tumor suppressive phenotype remain to be reconciled. In this study, we identified both fibroblast mediated PGE2 secretion and autocrine PGE2 signaling as a novel tumor promoting characteristic of these cells. Moreover, we identify a novel tumor promoting mechanism of these cells that is enhanced upon exposure to PGE2: the ability to secrete factors (such as IL-6) that are essential for the expansion of CD44^+^/CD24^−^/EpCAM^+^ breast cancer cells.

There exist a number of citations to substantiate the notion that fibroblasts harvested from resected breast tumor specimens are αSMA+ and promote tumor growth *in vivo* (reviewed by [11], [12]. These cells, commonly referred to as cancer associated fibroblasts (CAFs), lack specific markers to discriminate them from fibroblasts isolated from disease free tissues, because these normal fibroblast counterparts acquire the expression of αSMA when grown *in vitro*. Despite this, αSMA has become a widely used marker to discriminate these cells. We harvested both types of fibroblasts: fibroblasts from human breast tumors (CAFs), and fibroblasts from disease free reduction mammoplasty tissues. Both sources of fibroblasts expressed the fibroblast markers vimentin, prolyl-4-hydroxylase, and αSMA to unifying degrees *in vitro*, and both were largely negative for breast epithelium specific cytokeratins CK18 and CK14.

Despite similar fibroblast marker expression *in vitro*, both sources of fibroblasts had differences in their tumor promoting abilities *in vivo*. Because tumor-promoting ability of our fibroblast populations did not associate with any differences in marker expression examined, nor did it associate with tissue source, we sought for other characteristics of fibroblasts that may influence fibroblast tumor promoting abilities. Because fibroblasts isolated from dysplastic mouse skin support squamous cell carcinoma growth *in vivo* through pro-inflammatory cytokine secretion [15], we suspected that inflammatory mediators differ among the various patient derived fibroblasts, which ultimately influence their varying degrees of tumor promotion. Indeed, we found an association between tumor promoting ability *in vivo* and high PGE2 secretion *in vitro*. In addition, the ability to secrete PGE2 associates with the ability to expand CD44^+^/CD24^−^/EpCAM^+^ breast cancer cells.

Interestingly, PGE2 alone was not sufficient to confer the expansion of these cells, despite MCF7 expression of the prostanoid receptors. PGE2 enhanced the inherent tumor promoting abilities of fibroblasts as shown by *in vitro* tumorsphere assays, and *in vivo* MCF7 limiting dilution experiments. Our data suggest that this is largely due to the increased secretion of IL-6 in response to PGE2, consistent with previous reports [49]. The secretion of IL-6 by fibroblasts was necessary for expansion of CD44^+^/CD24^−^/EpCAM^+^ cells. Because this particular cell population has been shown to contribute to tumorsphere formation and seed tumor growth at limiting dilution [43], [50], we suspect that PGE2- mediated IL-6 secretion by fibroblasts was largely responsible for the increased MCF7 tumorsphere formation and tumor formation observed upon exposure to fibroblast CM.

Despite the requirement for IL-6 in mediating the tumor promoting abilities of fibroblasts, we found that IL-6 alone was not sufficient for expansion of CD44^+^/CD24^−^/EpCAM^+^ cells in the MCF7 cell line. This is in contrast to previous reports using different breast cancer cell lines [46], [51]. IL-6 has been implicated in a positive feedback loop that perpetuates cellular transformation through NFκB [46], which may drive the secretion of the other necessary cytokines (in addition to IL-6) that are required for the induction and maintenance of this aggressive cell state. We failed to see activation of NFκB in response to CM from various tissue-derived fibroblasts (data not shown), suggesting that this feedback mechanism may not be activated in these cells, or only activated in various subpopulations (i.e. CD44^+^/CD24^−^/EpCAM^+^ cells).

The basal level of IL-6 varied widely among patient derived breast fibroblasts, and also increased with passage number, consistent with previous reports using primary fibroblasts [52]. However, neither the basal secretion of IL-6 nor the induced secretion of IL-6 by PGE2 in fibroblasts correlated with fibroblast tumor promoting ability *in vivo* or their ability to expand CD44^+^/CD24^−^/EpCAM^+^ cells *in vitro*. This is consistent with the notion that IL-6 is required, but is not sufficient for expansion of CD44^+^/CD24^−^/EpCAM^+^ cells, nor is it sufficient for tumorsphere formation. In addition, although IGF-II was significantly induced by fibroblasts exposed to PGE2 more so than IL-6, we failed to observe a requirement for this cytokine in expansion of CD44^+^/CD24^−^/EpCAM^+^ cells (data not shown). In lieu of these data, we speculate that a certain stoichiometric balance of specific cytokines is required to promote the expansion of these cells. This stoichiometric balance may be obtained *in vivo* by secreted factors from tumor cells as well as inflammatory cells in the tumor microenvironment. It therefore follows that non-tumor promoting fibroblasts (i.e., those with very low PGE2 secretion; i.e. tissue sample I) perhaps lack the ability to activate certain pathways downstream of PGE2 that modulate the balance of cytokine secretion required for CD44^+^/CD24^−^/EpCAM^+^ cell expansion and tumor promoting ability *in vivo*. Further studies are needed to determine what precise signaling pathways are activated in stromal fibroblasts that activate Cox-2 expression and increase PGE2 and IL-6 secretion. Interestingly, recent studies have shown that PTEN loss in stromal fibroblasts significantly enhances fibroblast tumor promoting ability through upregulation of genes associated with ECM remodeling, wound healing and inflammatory responses [53]. Further investigation into the activation of the PI3K/Akt, Ras, and JNK pathways in stromal fibroblasts will elucidate which, if any, of these molecular players are upstream of PGE2 and IL-6 secretion in these cells.

Since PGE2 enhanced fibroblast secretion of IL-6, it is plausible that PGE2 also induces secretion of other cytokines yet to be identified that together in concert with IL-6 orchestrate the process of CD44^+^/CD24^−^/EpCAM^+^ cell expansion. For example, SDF1α is a growth factor secreted by myofibroblasts [10] that promotes breast cancer cell growth [54] as well as expansion of CD44^+^/CD24^−^ cells [55], and may be upregulated by PGE2 [56]. Also, IL-8 increases tumorsphere forming ability and the percentage of ALDEFLUOR-positive breast cancer cells, which enriches for cancer stem-like cell populations [57]. Further studies are needed to elucidate if these cytokines contribute to IL-6 mediated expansion of CD44^+^/CD24^−^/EpCAM^+^ cells.

In summary, our results indicate that the pro-inflammatory, tumor-promoting phenotype of fibroblasts correlates with the ability to secrete PGE2 and respond to PGE2 signaling. We were unable to identify differential expression of well-established markers of CAFs and tumor-promoting fibroblasts that correlated with their observed tumor promoting phenotypes. Moreover, we failed to identify a correlation between fibroblasts isolated from invasive carcinomas (either ER+ or ER−) and a high PGE2 secreting, tumor promoting phenotype, as compared to those isolated from reduction mammoplasty tissues. Our results suggest that tumor promoting fibroblasts secrete PGE2 and mediate autocrine PGE2 signaling, thereby activating the increased secretion of IL-6, which is required for expansion of breast cancer stem-like cells. In fact, our results suggest that the tumor promoting ability of fibroblasts associates moreso with PGE2 secretion and signaling than if these fibroblasts are derived from diseased tissues. The identification of specific markers for these tumor promoting fibroblasts would likely allow for the observation of more robust phenotypic discrepancies between various fibroblast tissue sources, and would possibly discriminate among patients that would likely benefit from tumor-associated stroma drug targeting strategies for use as adjuvant therapies in the treatment of human breast cancers.

## Supporting Information

Figure S1 **Characterization of patient derived fibroblasts from human breast tumor tissues and reduction mammoplasty tissues.** (**A**) Immunofluorescence results for the expression of mesenchymal markers vimentin and prolyl-4-hydroxylase (P4H), and myofibroblast/cancer associated fibroblast markers αSMA and Caveolin-1, in tissue-derived fibroblasts from patient samples A, C, E, I, M, N, and K. Nuclei are stained with DAPI. Scale bar, 50 µm. (**B**) Immunofluorescence results for the expression of the breast epithelial marker CK18 in tissue-derived fibroblasts from patient sample C, which showed high transcript levels of CK18 in Fig. 1A. MCF7 cells are a positive control.
(TIF)

Figure S2 **Tissue-derived fibroblasts express Cox-2 transcript and the prostanoid receptors.** (A) Quantitative RT-PCR for the relative levels of Cox-2 transcript in tissue derived fibroblasts from patient samples A, N, I, and F. MCF7 and SUM1315 breast cancer cells serve as negative and positive controls for Cox-2 expression, respectively. (B) Western blot for EP1, EP2, EP3 and EP4 expression in lysates extracted from tissue-derived fibroblasts (patients A and I), MCF7 and HCC1428 breast cancer cell lines.
(TIF)

Figure S3 **PGE2 enhances the ability of fibroblasts to expand CD44^+^/CD24^−^/ESA^+^ cells.** FACS dot plots of MCF7 cells treated with CM from tissue derived fibroblasts (patient samples I and A) treated with EtOH (vehicle) or 0.5 µM PGE2. Cell populations are gated first for EpCAM^+^, then for CD44^+^ and CD24^−^.
(TIF)

Figure S4 **Basal levels of IL-6 in various patient derived fibroblasts.** (**A**) Cytokine array results of CM from PGE2 and EtOH (vehicle) treated fibroblasts (from patient sample A). Data is plotted as a normalized fold induction over vehicle. (**B**) Quantification of the average basal levels of IL-6 secretion by various patient-derived fibroblasts using a human IL-6 ELISA. IL-6 secretion was normalized to the total number of fibroblasts present at the time of CM harvest.
(TIF)

Table S1 **Primer sequences used for quantitative RT-PCR.**
(PDF)
