# Supplementary figures and images for: Functional Heterogeneity of Breast Fibroblasts Is Defined by a Prostaglandin Secretory Phenotype that Promotes Expansion of Cancer-Stem Like Cells
Source: PLoS One. 2011 Sep 21;6(9):e24605. doi: 10.1371/journal.pone.0024605 (PMC3177828; doi:10.1371/journal.pone.0024605)

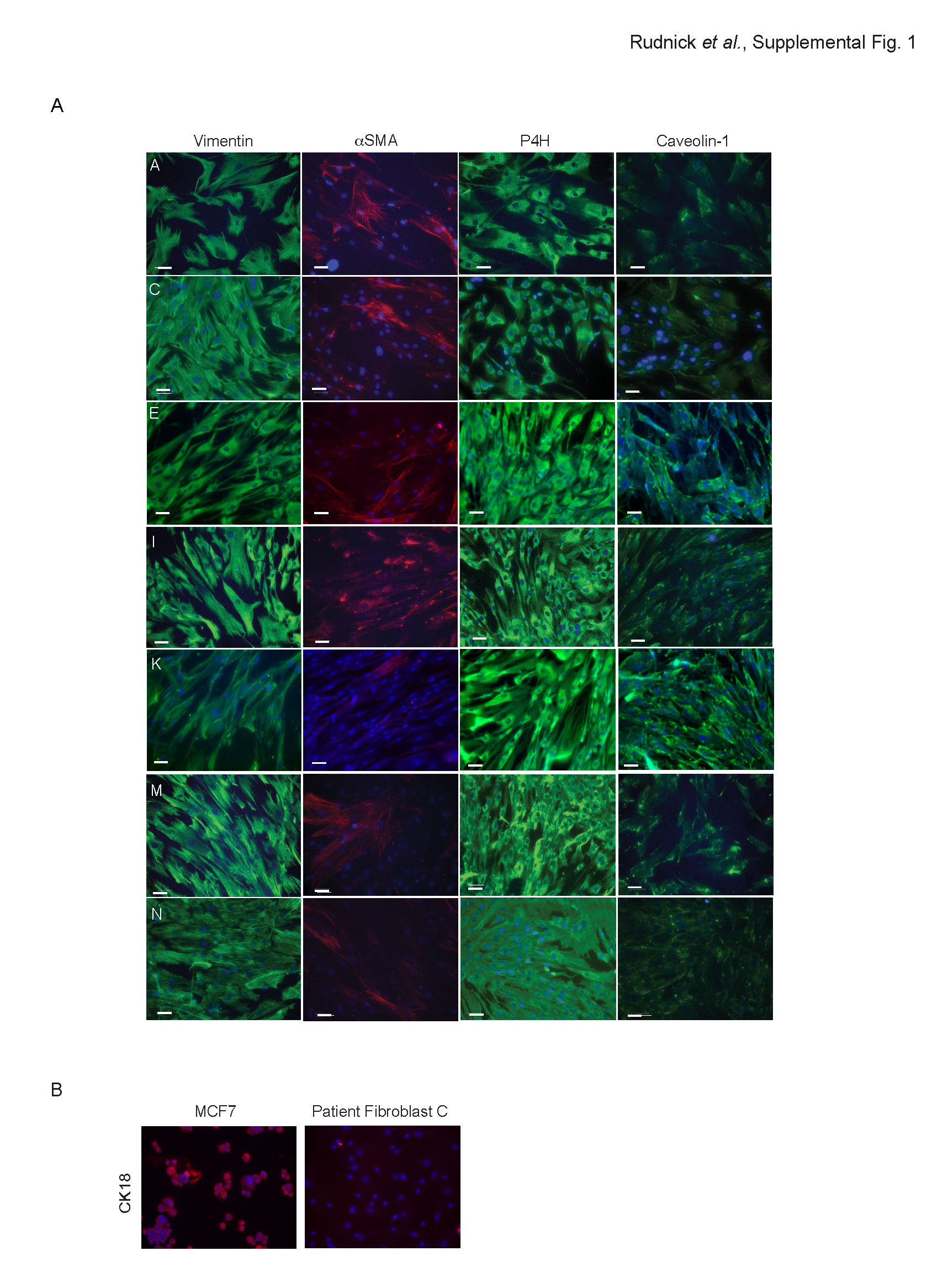

Supplement: Figure S1 — Characterization of patient derived fibroblasts from human breast tumor tissues and reduction mammoplasty tissues. (A) Immunofluorescence results for the expression of mesenchymal markers vimentin and prolyl-4-hydroxylase (P4H), and myofibroblast/cancer associated fibroblast markers αSMA and Caveolin-1, in tissue-derived fibroblasts from patient samples A, C, E, I, M, N, and K. Nuclei are stained with DAPI. Scale bar, 50 µm. (B) Immunofluorescence results for the expression of the breast epithelial marker CK18 in tissue-derived fibroblasts from patient sample C, which showed high transcript levels of CK18 in Fig. 1A. MCF7 cells are a positive control. (TIF) [file pone.0024605.s001.tif]

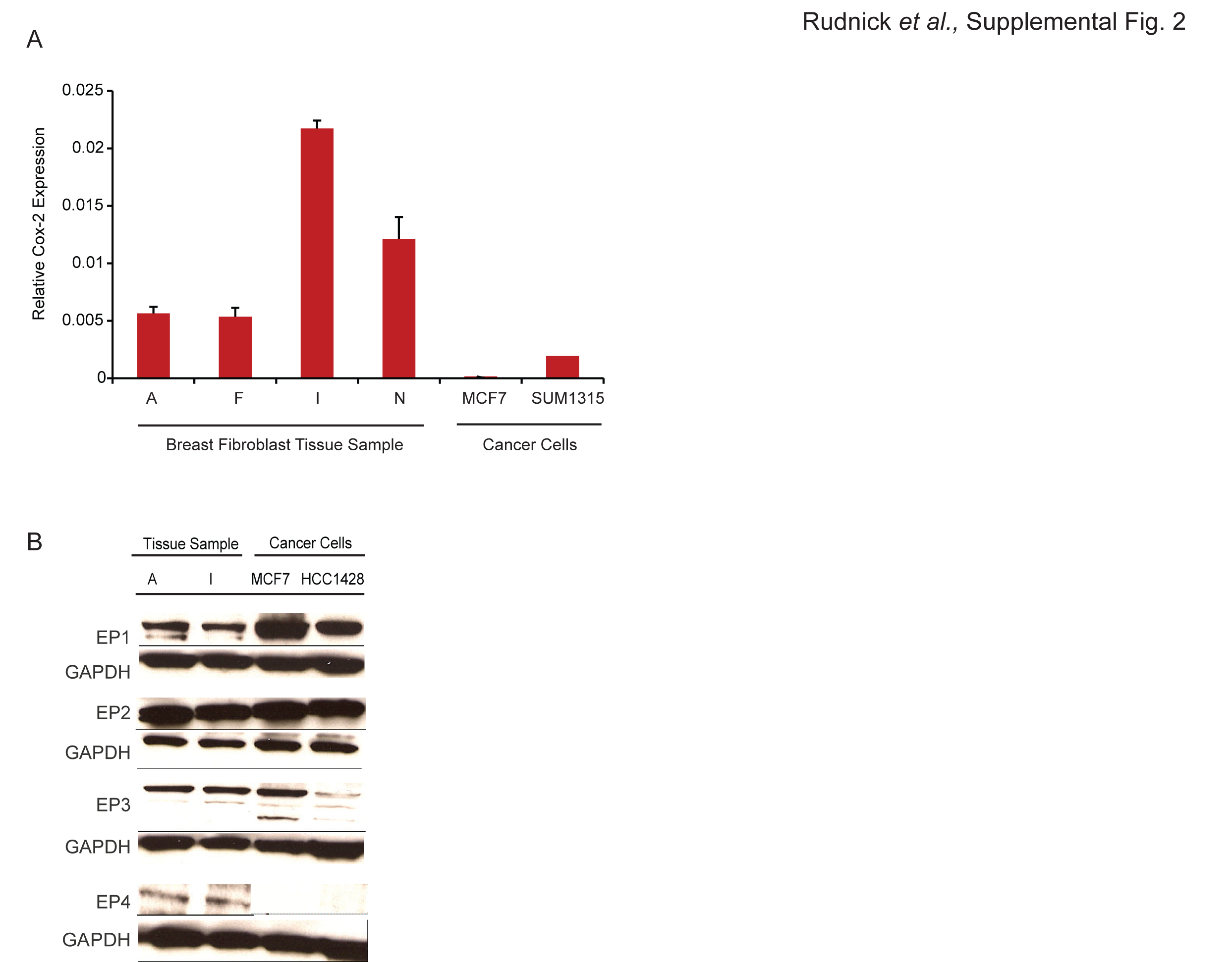

Supplement: Figure S2 — Tissue-derived fibroblasts express Cox-2 transcript and the prostanoid receptors. (A) Quantitative RT-PCR for the relative levels of Cox-2 transcript in tissue derived fibroblasts from patient samples A, N, I, and F. MCF7 and SUM1315 breast cancer cells serve as negative and positive controls for Cox-2 expression, respectively. (B) Western blot for EP1, EP2, EP3 and EP4 expression in lysates extracted from tissue-derived fibroblasts (patients A and I), MCF7 and HCC1428 breast cancer cell lines. (TIF) [file pone.0024605.s002.tif]

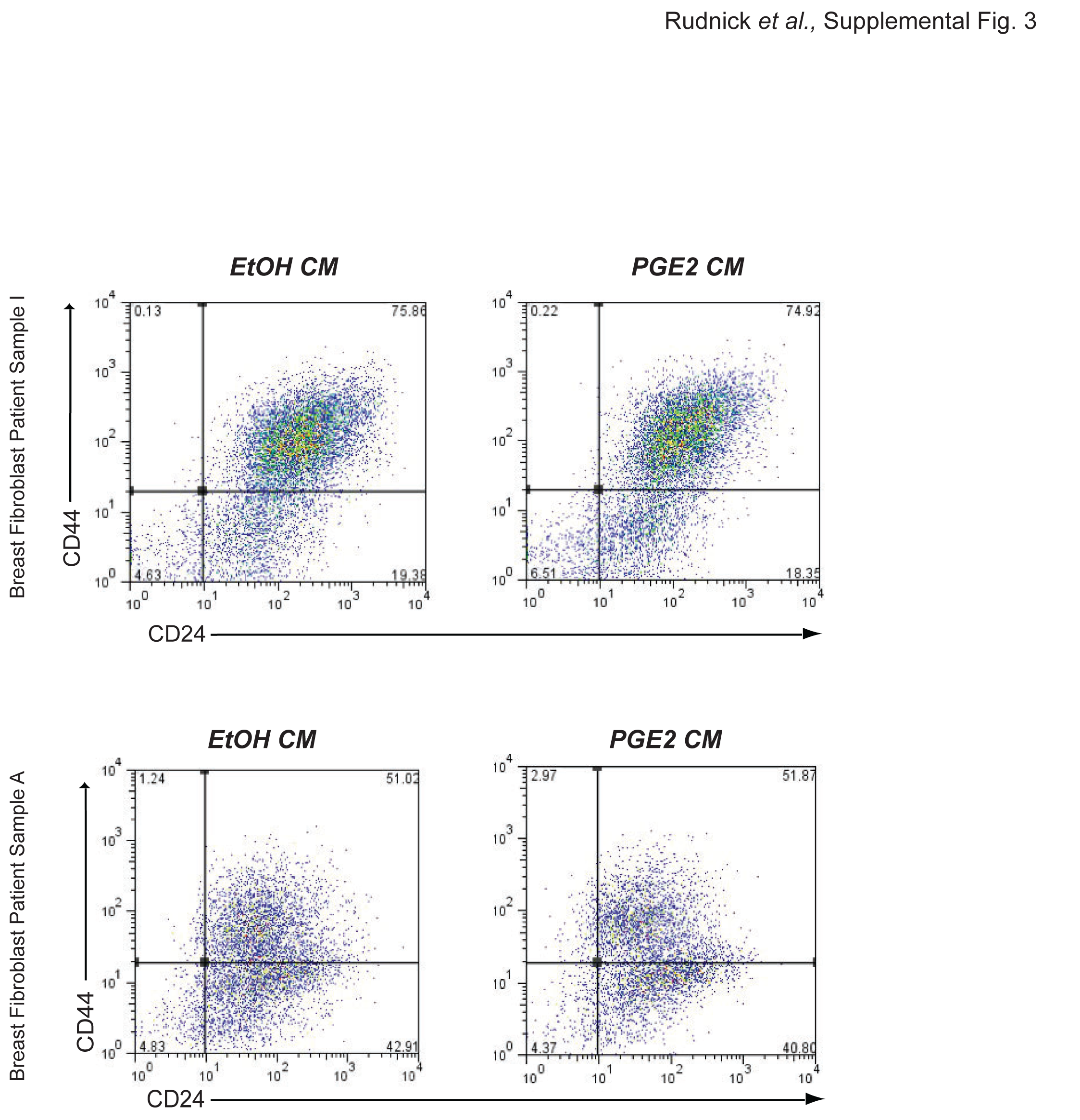

Supplement: Figure S3 — PGE2 enhances the ability of fibroblasts to expand CD44+/CD24−/ESA+ cells. FACS dot plots of MCF7 cells treated with CM from tissue derived fibroblasts (patient samples I and A) treated with EtOH (vehicle) or 0.5 µM PGE2. Cell populations are gated first for EpCAM+, then for CD44+ and CD24−. (TIF) [file pone.0024605.s003.tif]

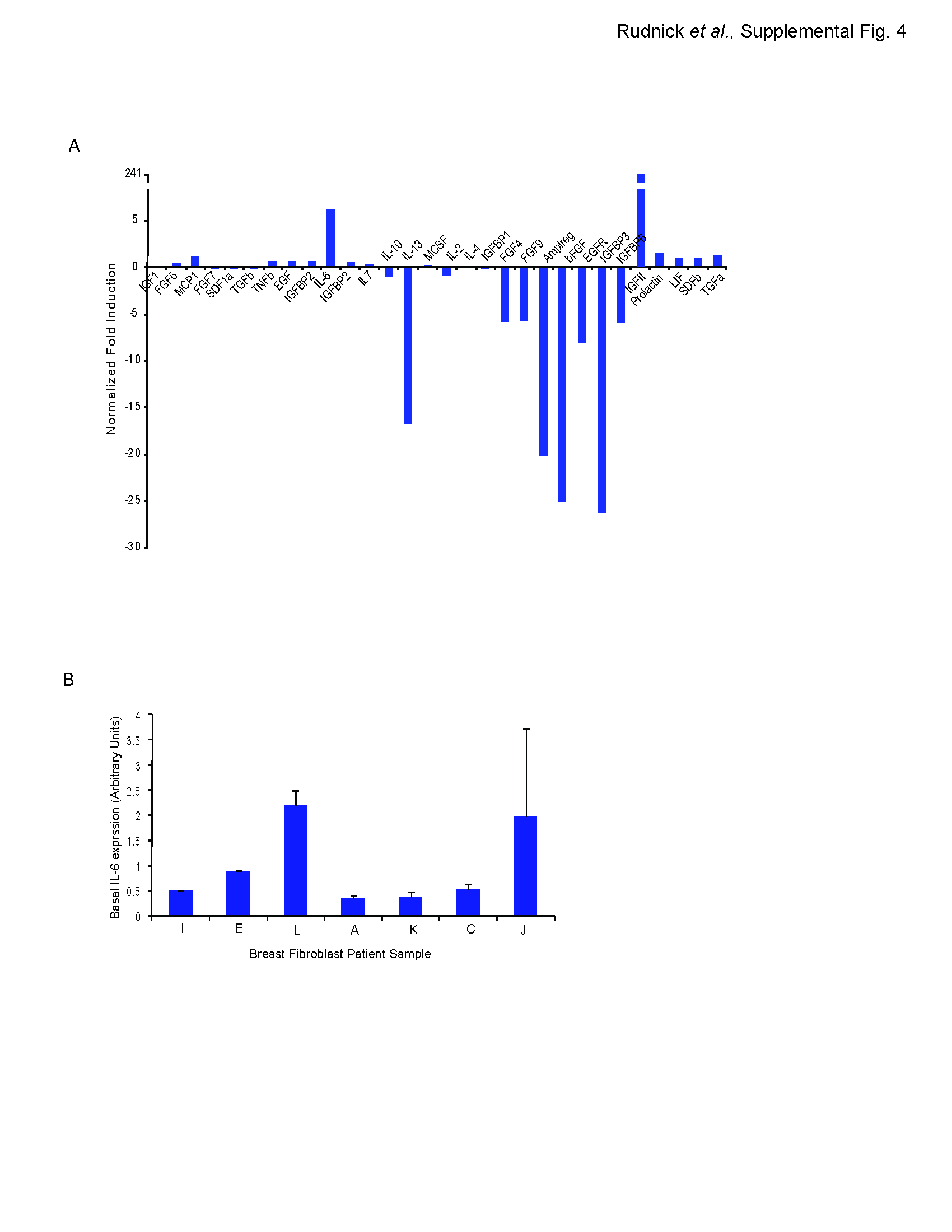

Supplement: Figure S4 — Basal levels of IL-6 in various patient derived fibroblasts. (A) Cytokine array results of CM from PGE2 and EtOH (vehicle) treated fibroblasts (from patient sample A). Data is plotted as a normalized fold induction over vehicle. (B) Quantification of the average basal levels of IL-6 secretion by various patient-derived fibroblasts using a human IL-6 ELISA. IL-6 secretion was normalized to the total number of fibroblasts present at the time of CM harvest. (TIF) [file pone.0024605.s004.tif]
