# Supplementary material for: Functional Heterogeneity of Breast Fibroblasts Is Defined by a Prostaglandin Secretory Phenotype that Promotes Expansion of Cancer-Stem Like Cells
Source: PLoS One. 2011 Sep 21;6(9):e24605. doi: 10.1371/journal.pone.0024605 (PMC3177828; doi:10.1371/journal.pone.0024605)
Supplement: Table S1 — Primer sequences used for quantitative RT-PCR. (PDF) [file pone.0024605.s005.pdf]

Supplementary Table 1: Primer sequences used for quantitative real time PCR.

Gene	Forward Primer	Reverse Primer
αSMA	5'CAGGGCTGTTTTCCCATCCT3'	5'GCCATGTTCTATCGGGTACTTC3'
FAP	5'AATGAGAGCACTCACACTGAAG3'	5'CCGATCAGGTGATAAGCCGTAAT3'
FSP	5'GATGAGCAACTTGGACAGCAA3'	5'CTGGGCTGCTTATCTGGGAAG3'
CK18	5'TGATGACACCAATATCACACGAC3'	5'TACCTCCACGGTCAACCCA3'
CK14	5'CATGAGTGTGGAAGCCGACAT3'	5'GCCTCTCAGGGCATTCATCTC3'
Cox2	5'TGAGCATCTACGGTTTGCTG3'	5'TGCTTGTCTGGAACAACTGC3'
GAPDH	5'GAGTCAACGGATTTGGTCGT3'	5'GACAAGCTTCCCGTTCTCAG3'
